# (*E*)-1-[4-(Hex­yloxy)phen­yl]-3-(4-hy­droxy­phen­yl)prop-2-en-1-one

**DOI:** 10.1107/S1600536810052086

**Published:** 2010-12-18

**Authors:** Zainab Ngaini, Siti Muhaini Haris Fadzillah, Hasnain Hussain, Ibrahim Abdul Razak, Hoong-Kun Fun

**Affiliations:** a Department of Chemistry, Faculty of Resource Science and Technology, Universiti Malaysia Sarawak, 94300 Kota Samarahan, Sarawak, Malaysia; b Department of Molecular Biology, Faculty of Resource Science and Technology, Universiti Malaysia Sarawak, 94300 Kota Samarahan, Sarawak, Malaysia; c X-ray Crystallography Unit, School of Physics, Universiti Sains Malaysia, 11800 USM, Penang, Malaysia

## Abstract

In the title compound, C_21_H_24_O_3_, inter­molecular O—H⋯O and C—H⋯O inter­actions form bifurcated acceptor bonds, generating *R*_2_^1^(6) ring motifs. These ring motifs link the mol­ecules into extended chains along [010]. The crystal structure is further stabilized by C—H⋯π inter­actions.

## Related literature

For the biological properties of chalcone derivatives, see: Bhat *et al.* (2005 ▶); Xue *et al.* (2004 ▶); Zhao *et al.* (2005 ▶); Lee *et al.* (2006 ▶). For related structures, see: Razak *et al.* (2009 ▶, 2009*a* ▶,*b* ▶); Ngaini, Fadzillah *et al.* (2009 ▶); Ngaini, Rahman *et al.* (2009 ▶). For details of hydrogen-bond motifs, see: Bernstein *et al.* (1995 ▶). For the stability of the temperature controller used in the data collection, see: Cosier & Glazer (1986 ▶). For bond-length data, see: Allen *et al.* (1987) ▶.
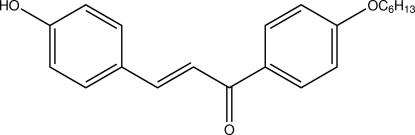


## Experimental

### 

#### Crystal data

C_21_H_24_O_3_
*M* *_r_* = 324.40
Monoclinic, 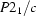

*a* = 10.2807 (2) Å
*b* = 16.6322 (2) Å
*c* = 11.4736 (2) Å
β = 113.439 (1)°
*V* = 1799.99 (5) Å^3^
*Z* = 4
Mo *K*α radiation
μ = 0.08 mm^−1^
*T* = 100 K
0.53 × 0.46 × 0.09 mm

#### Data collection

Bruker APEXII CCD area-detector diffractometer
Absorption correction: multi-scan (*SADABS*; Bruker, 2005 ▶) *T*_min_ = 0.960, *T*_max_ = 0.993
35735 measured reflections
5839 independent reflections
4743 reflections with *I* > 2σ(*I*)
*R*_int_ = 0.034

#### Refinement

*R*[*F* ^2^ > 2σ(*F* ^2^)] = 0.048
*wR*(*F* ^2^) = 0.135
*S* = 1.03
5839 reflections
219 parameters
H-atom parameters constrained
Δρ_max_ = 0.41 e Å^−3^
Δρ_min_ = −0.20 e Å^−3^

### 

Data collection: *APEX2* (Bruker, 2005 ▶); cell refinement: *SAINT* (Bruker, 2005 ▶); data reduction: *SAINT*; program(s) used to solve structure: *SHELXTL* (Sheldrick, 2008 ▶); program(s) used to refine structure: *SHELXTL*; molecular graphics: *SHELXTL*; software used to prepare material for publication: *SHELXTL* and *PLATON* (Spek, 2009 ▶).

## Supplementary Material

Crystal structure: contains datablocks global, I. DOI: 10.1107/S1600536810052086/fj2369sup1.cif

Structure factors: contains datablocks I. DOI: 10.1107/S1600536810052086/fj2369Isup2.hkl

Additional supplementary materials:  crystallographic information; 3D view; checkCIF report

## Figures and Tables

**Table 1:** Hydrogen-bond geometry (Å, °) *Cg*2 is the centroid of the C10–C15 ring.

*D*—H⋯*A*	*D*—H	H⋯*A*	*D*⋯*A*	*D*—H⋯*A*
O2—H2⋯O1^i^	0.84	1.86	2.694 (1)	175
C4—H4*A*⋯O1^i^	0.95	2.54	3.213 (1)	128
C16—H16*A*⋯*Cg*2^ii^	0.99	2.77	3.666 (1)	151

Symmetry codes: (i) 
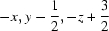
; (ii) 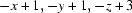.

**Fig. 1.**
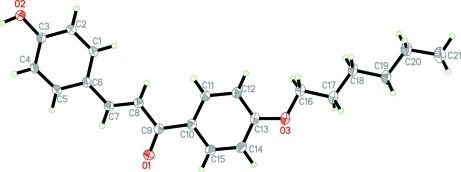
The molecular structure of the title compound, showing 50% probability displacement ellipsoids and the atom numbering scheme.

**Fig. 2.**
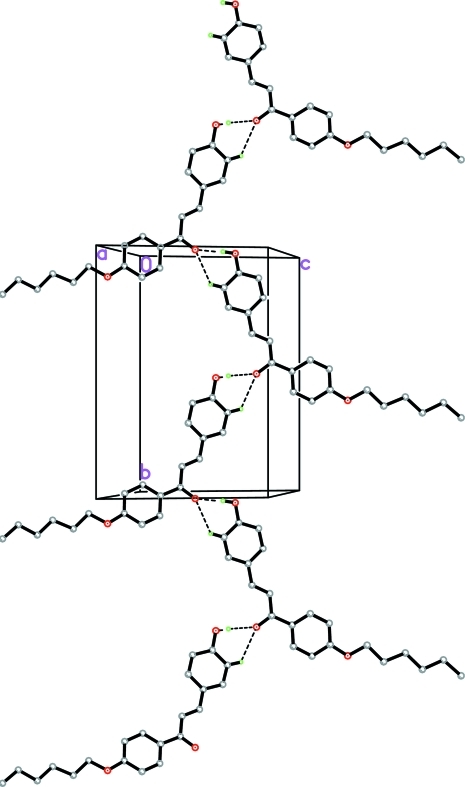
A molecular chain of the title compound along the b axis.

## supplementary crystallographic information

### Comment

The biological properties of chalcones derivatives have been extensively reported (Bhat *et al.*, 2005; Xue *et al.*, 2004; Lee *et al.*, 2006; Zhao *et al.*, 2005). Synthetic and naturally occurring chalcones have been widely studied and developed as one of the pharmaceutically important molecules. We have synthesized the title chalcone derivative, (I) which showed antimicrobial activity when tested against *E. coli* ATCC 8739 In this paper, we report the crystal structure of the title compound.

The bond lengths observed in the title compound (Fig.1) are comparable with previously reported values (Allen *et al.*, 1987). The conformation of the enone moiety (O1/C7—C9) is *s*-*cis* as shown by the value of -6.3 (2)° for O1—C9—C8—C7 torsion angle. The enone (O1/C7—C9) moiety adopts *s*-*cis* conformation with O2—C7—C8—C9 torsion angle being -6.3 (2)°. The dihedral angle between the mean plane through the enone moiety and the benzene rings is 5.1 (1)° for C1—C6 ring and 5.6 (1)° for C10—C15 ring. The two benzene rings make a dihedral angle of 7.8 (1)° between them.

The widening of C1—C6—C7 and C6—C7—C8 angles to 123.0 (1)° and 126.9 (1)° respectively, are the consequences of the close interatomic contact between H1A and H8A which is 2.20 Å. Similarly, the strain induced by short H8A···H11A contact, which is 2.09Å resulted in the opening of C9—C10—C11 angle to 123.8 (1)°. The distortion of angles, which is relative to what is predicted in terms of hybidization principles can also be observed in the related structures previously reported by Razak, Fun, Ngaini, Rahman *et al.* (2009), Razak, Fun, Ngaini, Fadzillah *et al.* (2009*a*,*b*), Ngaini, Fadzillah *et al.* (2009) and Ngaini, Rahman *et al.* (2009).

The conformation assumed by the zigzag alkoxyl tail is *trans*. Even though the torsion angle of C12—C13—O3—C16 is -1.3 (2)°, which shows that it is roughly coplanar with the attached benzene ring, the alkoxyl tail is actually twisted about the C18—C19 bond. Within the aliphatic chain, the C17—C18—C19—C20 torsion angle shows the value of -167.0 (1)°

In the crystal structure, intermolecular O2—H2···O1(-*x*,*y* - 1/2,-*z* + 3/2) and C4—H4A···O1(-*x*,*y* - 1/2,-*z* + 3/2) interactions form bifurcated acceptor bonds generating *R*^1^_2_(6) ring motifs (Bernstein *et al.*, 1995). These intermolecular interactions link the molecules into extended chains along the [0 1 0] direction. The crystal structure is further stabilized by C—H···π interactions (Table 1).

### Experimental

A mixture of 4-hydroxybenzaldehyde (1.22 g, 10 mmol), 4-hexyloxyacetophenone (2.20 g, 10 mmol) and KOH (2.02 g, 36 mmol) in 30 ml of methanol was heated at reflux for 24 h. The reaction was cooled to room temperature and acidified with cold diluted HCl (2 N). The resulting precipitate was filtered, washed and dried. After redissolving in hexane-ethanol mixture, followed by few days of slow evaporation, suitable crystals were collected for X-ray analysis.

### Refinement

All the C– and O-bound H atoms were positioned geometrically and refined using a riding model with C—H = 0.93–0.97Å and O—H = 0.84 Å. The *U*_iso_ values were constrained to be -1.5U_equ_ (methyl H and O atoms) and -1.2U_equ_ (other H atoms). The rotating model group was applied for the methyl group.

### Crystal data

**Table:**

C_21_H_24_O_3_	*F*(000) = 696
*M**_r_* = 324.40	*D*_x_ = 1.197 Mg m^−^^3^
Monoclinic, *P*2_1_/*c*	Mo *K*α radiation, λ = 0.71073 Å
Hall symbol: -P 2ybc	Cell parameters from 9960 reflections
*a* = 10.2807 (2) Å	θ = 2.3–31.1°
*b* = 16.6322 (2) Å	µ = 0.08 mm^−^^1^
*c* = 11.4736 (2) Å	*T* = 100 K
β = 113.439 (1)°	Plate, yellow
*V* = 1799.99 (5) Å^3^	0.53 × 0.46 × 0.09 mm
*Z* = 4	

### Data collection

**Table:**

Bruker APEXII CCD area-detector diffractometer	5839 independent reflections
Radiation source: sealed tube	4743 reflections with *I* > 2σ(*I*)
graphite	*R*_int_ = 0.034
π and ω scans	θ_max_ = 31.3°, θ_min_ = 2.2°
Absorption correction: multi-scan (*SADABS*; Bruker, 2005)	*h* = −15→14
*T*_min_ = 0.960, *T*_max_ = 0.993	*k* = −24→24
35735 measured reflections	*l* = −16→16

### Refinement

**Table:**

Refinement on *F*^2^	Primary atom site location: structure-invariant direct methods
Least-squares matrix: full	Secondary atom site location: difference Fourier map
*R*[*F*^2^ > 2σ(*F*^2^)] = 0.048	Hydrogen site location: inferred from neighbouring sites
*wR*(*F*^2^) = 0.135	H-atom parameters constrained
*S* = 1.03	*w* = 1/[σ^2^(*F*_o_^2^) + (0.0702*P*)^2^ + 0.4086*P*] where *P* = (*F*_o_^2^ + 2*F*_c_^2^)/3
5839 reflections	(Δ/σ)_max_ = 0.001
219 parameters	Δρ_max_ = 0.41 e Å^−^^3^
0 restraints	Δρ_min_ = −0.20 e Å^−^^3^

### Special details

**Table:**

Experimental. The crystal was placed in the cold stream of an Oxford Cryosystems Cobra open-flow nitrogen cryostat (Cosier & Glazer, 1986) operating at 100.0 (1) K.
Geometry. All e.s.d.'s (except the e.s.d. in the dihedral angle between two l.s. planes) are estimated using the full covariance matrix. The cell e.s.d.'s are taken into account individually in the estimation of e.s.d.'s in distances, angles and torsion angles; correlations between e.s.d.'s in cell parameters are only used when they are defined by crystal symmetry. An approximate (isotropic) treatment of cell e.s.d.'s is used for estimating e.s.d.'s involving l.s. planes.
Refinement. Refinement of *F*^2^ against ALL reflections. The weighted *R*-factor *wR* and goodness of fit *S* are based on *F*^2^, conventional *R*-factors *R* are based on *F*, with *F* set to zero for negative *F*^2^. The threshold expression of *F*^2^ > σ(*F*^2^) is used only for calculating *R*-factors(gt) *etc*. and is not relevant to the choice of reflections for refinement. *R*-factors based on *F*^2^ are statistically about twice as large as those based on *F*, and *R*- factors based on ALL data will be even larger.

### Fractional atomic coordinates and isotropic or equivalent isotropic displacement parameters (Å^2^)

**Table:**

	*x*	*y*	*z*	*U*_iso_*/*U*_eq_	
O1	0.15448 (9)	0.50631 (5)	0.96314 (7)	0.02611 (17)	
O2	−0.08883 (8)	0.02224 (4)	0.78756 (7)	0.02446 (16)	
H2	−0.1115	0.0146	0.7095	0.037*	
O3	0.37607 (9)	0.61122 (5)	1.53438 (7)	0.02772 (18)	
C1	0.03955 (11)	0.20205 (6)	0.97710 (9)	0.0226 (2)	
H1A	0.0805	0.2163	1.0645	0.027*	
C2	0.00156 (11)	0.12297 (6)	0.94356 (9)	0.0231 (2)	
H2A	0.0155	0.0836	1.0074	0.028*	
C3	−0.05759 (10)	0.10104 (6)	0.81509 (9)	0.02014 (19)	
C4	−0.08238 (10)	0.15987 (6)	0.72177 (9)	0.02078 (19)	
H4A	−0.1251	0.1457	0.6344	0.025*	
C5	−0.04439 (11)	0.23904 (6)	0.75716 (9)	0.02089 (19)	
H5A	−0.0618	0.2787	0.6931	0.025*	
C6	0.01911 (10)	0.26189 (6)	0.88518 (9)	0.01983 (18)	
C7	0.06282 (10)	0.34531 (6)	0.91739 (9)	0.02114 (19)	
H7A	0.0432	0.3814	0.8483	0.025*	
C8	0.12779 (10)	0.37647 (6)	1.03444 (9)	0.02112 (19)	
H8A	0.1482	0.3426	1.1062	0.025*	
C9	0.16801 (10)	0.46172 (6)	1.05398 (9)	0.01995 (18)	
C10	0.22628 (10)	0.49587 (6)	1.18407 (9)	0.01980 (19)	
C11	0.24298 (10)	0.45194 (6)	1.29304 (9)	0.02125 (19)	
H11A	0.2192	0.3964	1.2853	0.025*	
C12	0.29373 (11)	0.48771 (6)	1.41261 (10)	0.0228 (2)	
H12A	0.3043	0.4570	1.4856	0.027*	
C13	0.32897 (11)	0.56930 (6)	1.42408 (10)	0.0227 (2)	
C14	0.31631 (11)	0.61369 (6)	1.31623 (10)	0.0256 (2)	
H14A	0.3432	0.6687	1.3244	0.031*	
C15	0.26496 (11)	0.57768 (6)	1.19849 (10)	0.0239 (2)	
H15A	0.2555	0.6085	1.1258	0.029*	
C16	0.38894 (11)	0.57030 (7)	1.64912 (10)	0.0251 (2)	
H16A	0.4594	0.5263	1.6688	0.030*	
H16B	0.2966	0.5472	1.6399	0.030*	
C17	0.43689 (12)	0.63306 (7)	1.75310 (10)	0.0281 (2)	
H17A	0.3622	0.6745	1.7340	0.034*	
H17B	0.5234	0.6597	1.7539	0.034*	
C18	0.46809 (12)	0.59804 (7)	1.88375 (10)	0.0269 (2)	
H18A	0.5392	0.5546	1.9007	0.032*	
H18B	0.3803	0.5737	1.8838	0.032*	
C19	0.52330 (13)	0.65975 (7)	1.99042 (11)	0.0298 (2)	
H19A	0.4449	0.6959	1.9856	0.036*	
H19B	0.5975	0.6928	1.9792	0.036*	
C20	0.58473 (13)	0.61976 (7)	2.12078 (11)	0.0321 (2)	
H20A	0.5103	0.5862	2.1308	0.038*	
H20B	0.6628	0.5836	2.1247	0.038*	
C21	0.64050 (18)	0.67832 (10)	2.23034 (13)	0.0502 (4)	
H21A	0.6852	0.6485	2.3102	0.075*	
H21B	0.5619	0.7105	2.2330	0.075*	
H21C	0.7105	0.7139	2.2189	0.075*	

### Atomic displacement parameters (Å^2^)

**Table:**

	*U*^11^	*U*^22^	*U*^33^	*U*^12^	*U*^13^	*U*^23^
O1	0.0332 (4)	0.0223 (3)	0.0210 (4)	0.0006 (3)	0.0089 (3)	0.0028 (3)
O2	0.0315 (4)	0.0194 (3)	0.0214 (4)	−0.0027 (3)	0.0093 (3)	−0.0019 (3)
O3	0.0343 (4)	0.0242 (4)	0.0233 (4)	−0.0042 (3)	0.0100 (3)	−0.0070 (3)
C1	0.0279 (5)	0.0224 (4)	0.0164 (4)	−0.0004 (4)	0.0076 (4)	−0.0011 (3)
C2	0.0293 (5)	0.0217 (4)	0.0179 (4)	0.0000 (4)	0.0091 (4)	0.0016 (3)
C3	0.0213 (4)	0.0196 (4)	0.0199 (4)	−0.0004 (3)	0.0086 (4)	−0.0015 (3)
C4	0.0230 (4)	0.0220 (4)	0.0167 (4)	0.0003 (4)	0.0073 (4)	−0.0018 (3)
C5	0.0243 (4)	0.0214 (4)	0.0164 (4)	0.0012 (4)	0.0075 (4)	0.0011 (3)
C6	0.0220 (4)	0.0200 (4)	0.0177 (4)	0.0002 (3)	0.0081 (3)	−0.0011 (3)
C7	0.0225 (4)	0.0205 (4)	0.0202 (4)	0.0008 (3)	0.0083 (4)	0.0004 (3)
C8	0.0233 (4)	0.0191 (4)	0.0201 (4)	0.0002 (3)	0.0076 (4)	0.0002 (3)
C9	0.0185 (4)	0.0201 (4)	0.0201 (4)	0.0016 (3)	0.0064 (3)	−0.0002 (3)
C10	0.0179 (4)	0.0193 (4)	0.0205 (4)	0.0007 (3)	0.0058 (3)	−0.0008 (3)
C11	0.0214 (4)	0.0202 (4)	0.0213 (5)	−0.0028 (3)	0.0077 (4)	−0.0016 (3)
C12	0.0229 (4)	0.0239 (5)	0.0207 (5)	−0.0029 (4)	0.0078 (4)	−0.0017 (4)
C13	0.0209 (4)	0.0231 (5)	0.0226 (5)	−0.0015 (4)	0.0071 (4)	−0.0049 (4)
C14	0.0283 (5)	0.0183 (4)	0.0274 (5)	−0.0006 (4)	0.0082 (4)	−0.0025 (4)
C15	0.0265 (5)	0.0197 (4)	0.0234 (5)	0.0011 (4)	0.0076 (4)	0.0015 (4)
C16	0.0247 (5)	0.0269 (5)	0.0234 (5)	−0.0025 (4)	0.0093 (4)	−0.0054 (4)
C17	0.0294 (5)	0.0276 (5)	0.0256 (5)	−0.0027 (4)	0.0091 (4)	−0.0088 (4)
C18	0.0260 (5)	0.0267 (5)	0.0257 (5)	−0.0008 (4)	0.0077 (4)	−0.0071 (4)
C19	0.0310 (5)	0.0274 (5)	0.0271 (5)	0.0000 (4)	0.0074 (4)	−0.0078 (4)
C20	0.0324 (6)	0.0314 (6)	0.0295 (6)	0.0017 (5)	0.0093 (5)	−0.0061 (4)
C21	0.0633 (10)	0.0464 (8)	0.0306 (7)	−0.0042 (7)	0.0078 (6)	−0.0100 (6)

### Geometric parameters (Å, °)

**Table:**

O1—C9	1.2410 (12)	C12—C13	1.3971 (14)
O2—C3	1.3563 (12)	C12—H12A	0.9500
O2—H2	0.8400	C13—C14	1.4018 (15)
O3—C13	1.3545 (12)	C14—C15	1.3766 (15)
O3—C16	1.4406 (13)	C14—H14A	0.9500
C1—C2	1.3824 (14)	C15—H15A	0.9500
C1—C6	1.4039 (14)	C16—C17	1.5127 (14)
C1—H1A	0.9500	C16—H16A	0.9900
C2—C3	1.4009 (14)	C16—H16B	0.9900
C2—H2A	0.9500	C17—C18	1.5187 (16)
C3—C4	1.3971 (13)	C17—H17A	0.9900
C4—C5	1.3874 (14)	C17—H17B	0.9900
C4—H4A	0.9500	C18—C19	1.5235 (15)
C5—C6	1.4019 (13)	C18—H18A	0.9900
C5—H5A	0.9500	C18—H18B	0.9900
C6—C7	1.4598 (14)	C19—C20	1.5253 (17)
C7—C8	1.3435 (14)	C19—H19A	0.9900
C7—H7A	0.9500	C19—H19B	0.9900
C8—C9	1.4690 (13)	C20—C21	1.5113 (18)
C8—H8A	0.9500	C20—H20A	0.9900
C9—C10	1.4826 (14)	C20—H20B	0.9900
C10—C11	1.3985 (14)	C21—H21A	0.9800
C10—C15	1.4088 (14)	C21—H21B	0.9800
C11—C12	1.3925 (14)	C21—H21C	0.9800
C11—H11A	0.9500		
			
C3—O2—H2	109.5	C15—C14—H14A	119.9
C13—O3—C16	118.65 (8)	C13—C14—H14A	119.9
C2—C1—C6	121.61 (9)	C14—C15—C10	121.04 (10)
C2—C1—H1A	119.2	C14—C15—H15A	119.5
C6—C1—H1A	119.2	C10—C15—H15A	119.5
C1—C2—C3	119.80 (9)	O3—C16—C17	106.11 (9)
C1—C2—H2A	120.1	O3—C16—H16A	110.5
C3—C2—H2A	120.1	C17—C16—H16A	110.5
O2—C3—C4	122.97 (9)	O3—C16—H16B	110.5
O2—C3—C2	117.40 (9)	C17—C16—H16B	110.5
C4—C3—C2	119.64 (9)	H16A—C16—H16B	108.7
C5—C4—C3	119.74 (9)	C16—C17—C18	112.85 (9)
C5—C4—H4A	120.1	C16—C17—H17A	109.0
C3—C4—H4A	120.1	C18—C17—H17A	109.0
C4—C5—C6	121.57 (9)	C16—C17—H17B	109.0
C4—C5—H5A	119.2	C18—C17—H17B	109.0
C6—C5—H5A	119.2	H17A—C17—H17B	107.8
C5—C6—C1	117.59 (9)	C17—C18—C19	113.55 (9)
C5—C6—C7	119.41 (9)	C17—C18—H18A	108.9
C1—C6—C7	122.99 (9)	C19—C18—H18A	108.9
C8—C7—C6	126.90 (9)	C17—C18—H18B	108.9
C8—C7—H7A	116.6	C19—C18—H18B	108.9
C6—C7—H7A	116.6	H18A—C18—H18B	107.7
C7—C8—C9	121.52 (9)	C18—C19—C20	111.76 (10)
C7—C8—H8A	119.2	C18—C19—H19A	109.3
C9—C8—H8A	119.2	C20—C19—H19A	109.3
O1—C9—C8	121.19 (9)	C18—C19—H19B	109.3
O1—C9—C10	118.82 (9)	C20—C19—H19B	109.3
C8—C9—C10	119.99 (9)	H19A—C19—H19B	107.9
C11—C10—C15	118.10 (9)	C21—C20—C19	114.00 (11)
C11—C10—C9	123.83 (9)	C21—C20—H20A	108.8
C15—C10—C9	118.06 (9)	C19—C20—H20A	108.8
C12—C11—C10	121.48 (9)	C21—C20—H20B	108.8
C12—C11—H11A	119.3	C19—C20—H20B	108.8
C10—C11—H11A	119.3	H20A—C20—H20B	107.6
C11—C12—C13	119.29 (10)	C20—C21—H21A	109.5
C11—C12—H12A	120.4	C20—C21—H21B	109.5
C13—C12—H12A	120.4	H21A—C21—H21B	109.5
O3—C13—C12	124.81 (10)	C20—C21—H21C	109.5
O3—C13—C14	115.26 (9)	H21A—C21—H21C	109.5
C12—C13—C14	119.93 (9)	H21B—C21—H21C	109.5
C15—C14—C13	120.12 (10)		
			
C6—C1—C2—C3	0.63 (16)	C8—C9—C10—C15	179.28 (9)
C1—C2—C3—O2	177.98 (9)	C15—C10—C11—C12	1.21 (15)
C1—C2—C3—C4	−2.28 (15)	C9—C10—C11—C12	−177.74 (9)
O2—C3—C4—C5	−178.39 (9)	C10—C11—C12—C13	−0.09 (15)
C2—C3—C4—C5	1.89 (15)	C16—O3—C13—C12	−1.30 (15)
C3—C4—C5—C6	0.16 (15)	C16—O3—C13—C14	178.63 (9)
C4—C5—C6—C1	−1.76 (15)	C11—C12—C13—O3	178.38 (9)
C4—C5—C6—C7	177.29 (9)	C11—C12—C13—C14	−1.55 (15)
C2—C1—C6—C5	1.36 (15)	O3—C13—C14—C15	−177.87 (9)
C2—C1—C6—C7	−177.65 (10)	C12—C13—C14—C15	2.07 (16)
C5—C6—C7—C8	−178.26 (10)	C13—C14—C15—C10	−0.93 (16)
C1—C6—C7—C8	0.73 (16)	C11—C10—C15—C14	−0.70 (15)
C6—C7—C8—C9	179.19 (9)	C9—C10—C15—C14	178.32 (9)
C7—C8—C9—O1	−6.32 (15)	C13—O3—C16—C17	−177.53 (9)
C7—C8—C9—C10	173.83 (9)	O3—C16—C17—C18	−175.01 (9)
O1—C9—C10—C11	178.37 (9)	C16—C17—C18—C19	177.23 (9)
C8—C9—C10—C11	−1.77 (14)	C17—C18—C19—C20	−166.95 (10)
O1—C9—C10—C15	−0.58 (14)	C18—C19—C20—C21	−179.71 (11)

### Hydrogen-bond geometry (Å, °)

**Table:**

Cg2 is the centroid of C10–C15 ring.

**Table:**

*D*—H···*A*	*D*—H	H···*A*	*D*···*A*	*D*—H···*A*
O2—H2···O1^i^	0.84	1.86	2.694 (1)	175
C4—H4A···O1^i^	0.95	2.54	3.213 (1)	128
C16—H16A···Cg2^ii^	0.99	2.77	3.666 (1)	151

Symmetry codes: (i) −*x*, *y*−1/2, −*z*+3/2; (ii) −*x*+1, −*y*+1, −*z*+3.

## References

[bb15] Allen, F. H., Kennard, O., Watson, D. G., Brammer, L., Orpen, A. G. & Taylor, R. (1987). *J. Chem. Soc. Perkin Trans. 2*, pp. S1–19.

[bb1] Bernstein, J., Davis, R. E., Shimoni, L. & Chang, N.-L. (1995). *Angew. Chem. Int. Ed. Engl.* 34, 1555–1573.

[bb2] Bhat, B. A., Dhar, K. L., Puri, S. C., Saxena, A. K., Shanmugavel, M. & Qazi, G. N. (2005). *Bioorg. Med. Chem. Lett.* **15**, 3177–3180. 10.1016/j.bmcl.2005.03.121 15893928

[bb3] Bruker (2005). *APEX2* , *SAINT* and *SADABS* Bruker AXS Inc., Madison, Wisconsin, USA.

[bb4] Cosier, J. & Glazer, A. M. (1986). *J. Appl. Cryst.* **19**, 105–107.

[bb5] Lee, Y. S., Lim, S. S., Shin, K. H., Kim, Y. S., Ohuchi, K. & Jung, S. H. (2006). *Biol. Pharm. Bull.* **29**, 1028–1031. 10.1248/bpb.29.1028 16651739

[bb6] Ngaini, Z., Fadzillah, S. M. H., Rahman, N. I. A., Hussain, H., Razak, I. A. & Fun, H.-K. (2009). *Acta Cryst.* E**65**, o879–o880. 10.1107/S1600536809010617 PMC2968884 21582590

[bb7] Ngaini, Z., Rahman, N. I. A., Hussain, H., Razak, I. A. & Fun, H.-K. (2009). *Acta Cryst.* E**65**, o889–o890. 10.1107/S1600536809010848 PMC2968804 21582598

[bb8] Razak, I. A., Fun, H.-K., Ngaini, Z., Fadzillah, S. M. H. & Hussain, H. (2009*a*). *Acta Cryst.* E**65**, o881–o882. 10.1107/S160053680901054X PMC2969075 21582591

[bb9] Razak, I. A., Fun, H.-K., Ngaini, Z., Fadzillah, S. M. H. & Hussain, H. (2009*b*). *Acta Cryst.* E**65**, o1133–o1134. 10.1107/S1600536809014925 PMC2977806 21583943

[bb10] Razak, I. A., Fun, H.-K., Ngaini, Z., Rahman, N. I. A. & Hussain, H. (2009). *Acta Cryst.* E**65**, o1092–o1093. 10.1107/S160053680901441X PMC2977771 21583907

[bb11] Sheldrick, G. M. (2008). *Acta Cryst.* A**64**, 112–122. 10.1107/S0108767307043930 18156677

[bb12] Spek, A. L. (2009). *Acta Cryst.* D**65**, 148–155. 10.1107/S090744490804362X PMC2631630 19171970

[bb13] Xue, C. X., Cui, S. Y., Liu, M. C., Hu, Z. D. & Fan, B. T. (2004). *Eur. J. Med. Chem.* **39**, 745–753. 10.1016/j.ejmech.2004.05.009 15337287

[bb14] Zhao, L. M., Jin, H. S., Sun, L. P., Piao, H. R. & Quan, Z. S. (2005). *Chem. Lett.* **15**, 5027–5029. 10.1016/j.bmcl.2005.08.039 16169724

